# Leveraging Free Public Use Data for Aging and Life Course Research

**DOI:** 10.1093/geroni/igab046.1921

**Published:** 2021-12-17

**Authors:** Lara Cleveland, Kathleen Cagney

## Abstract

This symposium will showcase life course and aging research that is possible using freely available integrated census and survey data available via IPUMS. This session is organized by the Network for Data-Intensive Research on Aging (NDIRA) initiative at the University of Minnesota’s Life Course Center. NDIRA seeks to build and support an interdisciplinary community of scientists leveraging powerful data resources in innovative ways to understand health outcomes at older ages, as well as the demography and economics of aging. The session features papers that illustrate how to examine aging-related topics including health at older ages, work and socioeconomic conditions, and living conditions with a common thread of examining heterogeneity within groups. These papers all leverage freely available census and nationally-representative survey data, highlighting the potential value of these data for studying aging and the life course. By combining papers on an array of topics from a variety of data sources, this symposium highlights exemplar papers that demonstrate the types of novel research possible using public use census and survey data that NDIRA seeks to foster.

